# Renal amyloidosis in Whipple disease: a case report

**DOI:** 10.4076/1757-1626-2-8444

**Published:** 2009-09-17

**Authors:** Stanislaw Niemczyk, Ewa Filipowicz, Lukasz Wozniacki, Janusz Grochowski, Leszek Zaleski, Agnieszka Grzejszczak, Agnieszka Perkowska Ptasinska, Lukasz Koperski, Joanna Matuszkiewicz Rowinska

**Affiliations:** 1 Department of Nephrology, Dialysis Therapy and Internal Medicine, Medical University of Warsaw1a Banacha Street, 02-097 WarsawPoland; 2 Department of Gastroenterology, Diabetes and Internal Medicine, Medical University of Warsaw1a Banacha Street; 02-097 WarsawPoland; 3 Department of Pathology, Medical University of Warsaw5 Chalubinskiego Street, 02-004 WarszawaPoland; 4 Department of Nephrology, Institute of Transplantology, Medical University of Warsaw59 Nowogrodzka Street, 02-006 WarszawaPoland

## Abstract

**Introduction:**

Whipple disease is a rare systemic infection caused by *Tropheryma whippelii* that usually manifests with joint pain, weight loss, diarrhoea and abdominal pain. However, in some cases the infection may involve other organs and tissues.

**Case presentation:**

We report on a 44-year-old man with Whipple disease which led to renal amyloidosis and end-stage renal failure. In this case, the patient was diagnosed with Whipple disease and commenced on a 12-month trimetoprime-sulfametoxasole therapy with good result. Six months after cessation of therapy the patient was readmitted to hospital due to signs of renal failure. An urgent kidney biopsy was performed which revealed secondary amyloidosis. Despite intensive immunosuppressive treatment, renal parameters gradually deteriorated and haemodialysis was started eventually. Three months later the patient’s general condition dramatically worsened with bloody diarrhoea, bilious vomiting and progressive malnutrition. The repeated endoscopic examination confirmed severe recurrence of Whipple disease. Ceftriaxone and total parenteral nutrition was started what greatly improved patient’s state.

**Conclusions:**

To our knowledge based on systematic review, this is the first case report on Whipple disease complicated by secondary amyloidosis and kidney failure maintained on permanent renal replacement therapy. It is strongly suspected that the use of immunosuppressive treatment in such cases may exacerbate the course of Whipple disease and cause life-threatening complications.

## Introduction

Whipple disease is a rare chronic multisystem disorder caused by a recently identified pathogen *Tropheryma whipelii* belonging to order *Actinomycetes* [1]. The disease mainly involves the gastrointestinal tract, but may also affect central nervous system (up to 40% of cases), heart, liver, lungs, bone marrow, skin, abdominal and peripheral lymph nodes, as well as other tissues [2-4]. Clinically, the classic symptoms of Whipple disease include joint pain, weight loss, diarrhoea and abdominal pain. However, some patients may report less specific ailments as a result of other organs involvement. Herein, we report a case of a young man with Whipple disease which led to renal amyloidosis and loss of kidney function requiring haemodialysis. We also indicate the potential negative role of immunosuppressive therapy on the course of Whipple disease.

## Case presentation

A 44-year-old Polish Caucasian male, farmer, was admitted to hospital due to 3-month history of high-grade fevers, loss of weight, diarrhoea and joint pain. Physical examination revealed swollen and reddened major joints of upper and lower extremities. Laboratory and imaging examinations excluded rheumatoid arthritis, ulceration and neoplasm. Upper endoscopy revealed non-specific inflammation of duodenum. Biopsy stains were not contributory. In view of signs and symptoms a strong suspicion of Crohn’s disease was made and the patient was commenced on prednisone 30 mg per day with a good result.

Several months later diarrhoea relapsed, malaise and loss of weight was noticeable. Laboratory tests were unremarkable (including creatinine level) except for hemoglobin which was below reference value (10 g/dL). Upper endoscopy was repeated and duodenum biopsy samples were collected. Histochemical stains showed periodic acid-Schiff (PAS)-positive particles in mucosa, which led to a diagnosis of Whipple disease. Trimetoprime-sulfametoxasole in dose of 960 mg twice per day was introduced. The patient was recommended to continue the treatment for at least one year. At that time diarrhoea subsided, test for proteinuria was negative and all other renal parameters were within normal limits.

**Figure 1.**
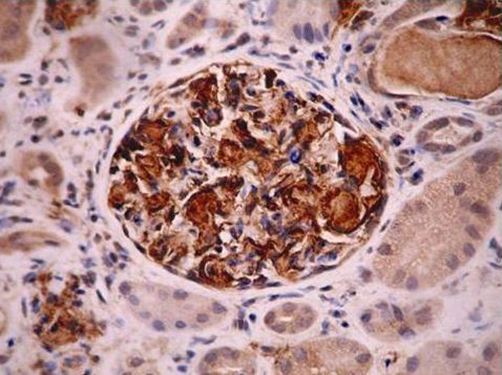
Immunohistochemical staining of biopsy specimen - glomerulus with amyloid-A (AA) deposits.

One and half year later the patient was readmitted to the hospital due to lower limbs oedema, weakness and anorexia. Laboratory tests revealed nephritic proteinuria (4-6 g/day), increased concentration of creatinine (1.2 mg/dL) and erythrocyte sedimentation rate (115 mm), iron deficiency anemia, decreased glomerular filtration rate (GFR) (52 ml/min) and normal urea (42 mg/dL). A kidney biopsy was performed which showed secondary amyloidosis (Figure 1). Computed tomography scans revealed enlargement of mesenteric and retroperitoneal lymph nodes with high amount of lipids which was suggestive of Whipple disease.

The patient received 1000 mg of cyclophosphamide, 20 mg of prednisone, and trimetoprime-sulfametoxasole. A month later he received second pulse of cyclophosphamide in dose of 400 mg. At this stage, creatinine concentration was 2.7 mg/dL and urea 170 mg/dL. Within next 3 weeks renal parameters markedly deteriorated and renal replacement therapy was started.

Three months later the patient was hospitalized again due to bloody diarrhoea, bilious vomiting, progressive malnutrition, anemia requiring erythrocyte transfusion and intradialysis hypotension. He denied having fever, abdominal pain or rectal tenesmus. On admission the patient was in poor general condition with orthostatic hypotension of 60/0 mm Hg and blood pressure of 80/50 mm Hg in recumbency. Heart rate was 64/min, body temperature was 36.6°C, BMI was 20. Muscular atrophy and malnutrition were easily seen. At this stage the results of basic laboratory tests were as follows: hemoglobin level 8.5 g/dL, hematocrit 25%, red blood cells counts 2.8 × 10^6^/µL, white blood cells counts 5.67 × 10^3^/µL, lymphocyte counts 16%, serum total protein 3.1 g/dL, albumin 1.6 g/dL, CRP 43 mg/dL and ferritin 1839 ng/mL. Additional biochemical tests revealed CD8 deficiency and abnormal CD4/CD8 ratio, decreased concentration of IgA, IgM and IgG, elevated level of IgE, reduced concentration of FT3 and testosterone, normal value of TSH, cortisol, prolactin and fasting insulin. Urine analysis detected proteinuria of 2.9 g/dL.

**Figure 2.**
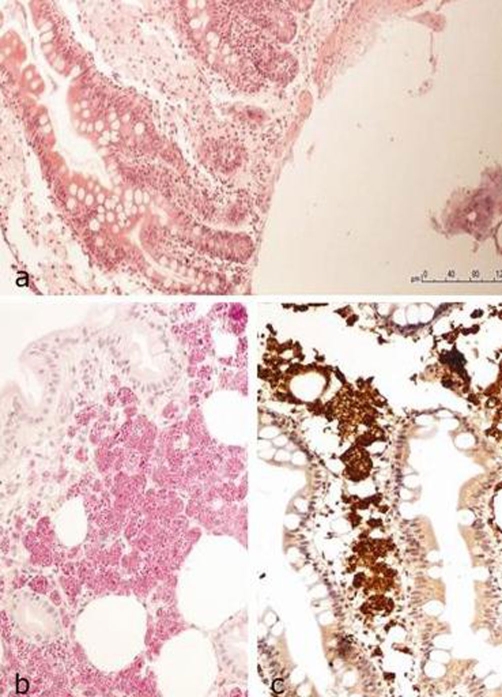
Duodenum mucosa with extended intestinal villi **(A)** and numerous PAS-positive macrophages **(B)**. Immunohistochemical staining for CD68 - markers of macrophages **(C)**.

Upper endoscopy was performed, which showed sever inflammation of post-bulbar duodenum with diffuse fibrinous erosions. Endoscopic examination also revealed fistula 60 cm from incisors. Histologic staining of collected biopsy samples revealed blood extravasation with large number of PAS-positive macrophages (Figure 2). This pathomorphologic picture was suggestive of Whipple disease. Subsequently, gastrointestinal series were performed which did not confirm the presence of fistula.

On the basis of above findings a diagnosis of severe exacerbation of Whipple disease was established. The patient was started on ceftriaxone (1 × 2.0 g per day) and total parenteral nutrition. Within few days of treatment diarrhoea and hypotension subsided. Repeated gastroscopy showed tremendous improvement of lesions. In view of difficult to manage nephritic proteinuria (daily protein depletion of 10-20 g), we decided to perform pharmacological nephrectomy and increase dehydration during dialysis. The patient made excellent progress and was discharged home 2 weeks later. He was recommended to receive doxycyclin in dose of 2 × 100 mg for 12 months. During the therapy daily diuresis decreased from 2000 ml to 350 ml.

**Figure 3.**
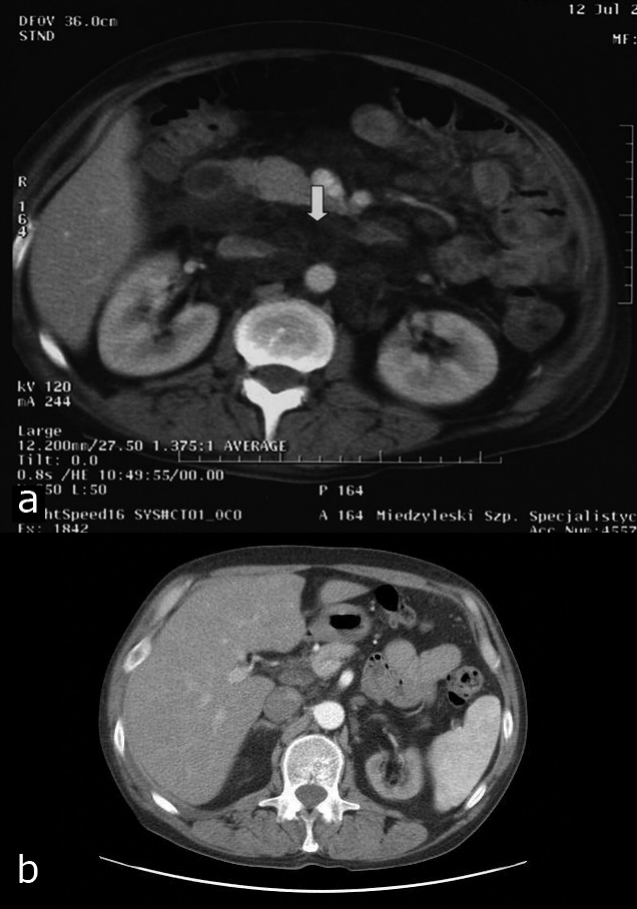
Computed tomography scans of the abdomen shows enlargement of mesenteric and retroperitoneal lymph nodes with high amount of lipids (exacerbation of Whipple disease) **(A)**. Marked reduction of lymph nodes on one-year follow-up **(B)**.

At 12-month follow-up, the patient presented in good general condition with normal appetite and without diarrhoea. He gained 5 kg (BMI increased to 21.8) and his exercise tolerance improved so that he could get back to work. Currently, the patient is on renal replacement therapy 3 times a week with the use of arteriovenous fistula. He receives doxycyclin regularly. Control computed tomography scans showed marked reduction of abdominal lymph nodes (maximum 2 cm in diameter) (Figure 3).

## Discussion

Whipple disease is a rare chronic multisystemic bacterial disease that is potentially fatal, but responds dramatically to antibiotic treatment, thus early diagnosis is mandatory. Although the first description of Whipple disease comes from 1907, this type of bowel infection is still a diagnostic challenge for both clinicians and pathologists.

Clinical symptoms like diarrhoea, weight loss, abdominal and joint pain are not pathognomonic for Whipple disease and require differentiation with a long list of other disorders, especially if neurological or ophthalmologic manifestations occur. The basis of histopathological recognition is the presence of foamy macrophages with periodic acid-Schiff-positive inclusions in tissues such as lamina propria [5]. Microbiological confirmation of diagnosis is problematic. Culture of *Tropheryma whipplei* has been established only once, in human fibroblast cell lines from a heart valve inoculum what makes it unavailable in clinical practice. Molecular-based diagnostic techniques, like nucleotide sequencing and amplification by reverse transcriptase-polymerase chain reaction (rtPCR) of bacterial S ribosomal RNA, although highly sensitive, are less specific, expensive and not available in Poland. [6]. New diagnostic tools involving isolation of bacteria from contaminated intestinal biopsies and immunohistological detection need to be developed.

The case described here represents a very uncommon presentation of Whipple disease, characterized by renal amyloidosis leading to end stage renal failure. To recapitulate available reports on amyloidosis in the course of Whipple disease a PubMed literature search from 1960 to January 2009 was performed using the following search strategy: (amyloidosis or amyloid or (renal failure) or (end stage renal disease) or (end stage renal failure) or dialysis or (renal replacement therapy)) and ((Whipple disease) or (Whipple’s disease) or Whipple or Whipple’s or (Whipple’s arthritis) or (Tropheryma whipplei) or (Tropheryma) or (lipodystrophy)).

In addition, references from identified publications and review papers on Whipple disease were also searched to identify other reported cases. No type of article or language restriction was set. Overall 107 papers on Whipple disease were identified, of which 2 reports concerned deterioration of renal function [7-8], 1 report generalized amyloidosis [9] and 5 reports renal amyloidosis (confirmed by histopathology) [10-14]. None of the patients with renal amyloidosis required dialysis or there was lack of such data.

## Conclusion

In presented case amyloidosis affected kidneys (confirmed by renal biopsy) and manifested as nephritic syndrome and rapidly progressive loss of renal function. The severe relapse of the disease occurred after completion of 12-month treatment with trimethoprim-sulfamethoxazole. Trimethoprim-sulfamethoxazole is regarded as a first-line antibiotic for Whipple disease but its efficacy may decrease during long treatment due to mutations in the target gene of sulfamethoxazole [15]. It cannot be excluded that such failure treatment occurred in this case. Another important issue is the implementation of immunosuppressive treatment (cyclophosphamide and prednisone) due to renal amyloidosis what probably led to severe relapse of Whipple disease. Such negative association between Whipple disease and immunosuppressive medications was suggested previously by several authors [16-19]. After administration of antibiotic therapy the patient’s general condition improved dramatically, however, kidneys injury was irreversible and the patient required renal replacement therapy. To our knowledge based on systematic review, this is the first case report on Whipple disease exacerbated by immunosuppressive treatment, complicated by renal amyloidosis and severe impairment of renal function.
